# High pressure minerals in the Château-Renard (L6) ordinary chondrite: implications for collisions on its parent body

**DOI:** 10.1038/s41598-018-28191-6

**Published:** 2018-06-29

**Authors:** Ioannis Baziotis, Paul D. Asimow, Jinping Hu, Ludovic Ferrière, Chi Ma, Ana Cernok, Mahesh Anand, Dan Topa

**Affiliations:** 1 Department of Natural Resources Management and Agricultural Engineering, Agricultural Univ. of Athens, Iera Odos 75, 11855 Athens, Greece; 2 California Institute of Technology, Division of Geological and Planetary Sciences, Pasadena, California, 91125 USA; 3 Natural History Museum, Burgring 7, A-1010 Vienna, Austria; 4 Planetary and Space Sciences, The Open University, Milton Keynes, MK7 6AA United Kingdom; 5 Department of Earth Sciences, The Natural History Museum, London, SW7 5BD United Kingdom

## Abstract

We report the first discoveries of high-pressure minerals in the historical L6 chondrite fall Château-Renard, based on co-located Raman spectroscopy, scanning electron microscopy (SEM) with energy-dispersive X-ray spectroscopy and electron backscatter diffraction, electron microprobe analysis, and transmission electron microscopy (TEM) with selected-area electron diffraction. A single polished section contains a network of melt veins from ~40 to ~200 μm wide, with no cross-cutting features requiring multiple vein generations. We find high-pressure minerals in veins greater than ~50 μm wide, including assemblages of ringwoodite + wadsleyite, ringwoodite + wadsleyite + majorite-pyrope_ss_, and ahrensite + wadsleyite. In association with ahrensite + wadsleyite at both SEM and TEM scale, we find a sodic pyroxene whose Raman spectrum is indistinguishable from that of jadeite but whose composition and structure are those of omphacite. We discuss constraints on the impact record of this meteorite and the L-chondrites in general.

## Introduction

Meteorites, especially ordinary chondrites, preserve a record of impact events due to (possibly multiple) collisions among their parent asteroids^1–4^. As such, meteorites showing evidence for shock and melting (i.e., impact metamorphism) at various scales (from cm down to nm) provide an opportunity to explore collision events and to constrain parameters including the pressure (*P*) – temperature (*T*) – time (*t*) conditions of impact metamorphism and hence the relative velocity, size, and density of impactors and targets. Shock parameters can be inferred from the occurrence and textures of high-pressure (HP) minerals, many of them are polymorphs of common low-pressure minerals^5–12^. HP minerals in ordinary chondrites are mostly found in association with melt veins (MVs). Therefore, relating the transformation and back-transformation kinetics of HP phases to MV thermal modeling provides important clues regarding the cooling history during and after the passage of the shock pulse^13,14^.

A number of highly-shocked L6 ordinary chondrites have been previously studied, with Tenham being perhaps the most intensively investigated^3,15–17^. In these meteorites, a variety of HP minerals have been reported including, in alphabetical order: ahrensite (Fe-analogue of ringwoodite), akimotoite (ilmenite-structured polymorph of enstatite), bridgmanite (perovskite-structured polymorph of enstatite), hemleyite (Fe-analogue of akomotoite), jadeite (monoclinic *C2/c* sodic pyroxene), lingunite (hollandite structured sodic plagioclase), majorite (garnet-structured polymorph of enstatite), ringwoodite (spinel-structured polymorph of olivine), stishovite (rutile-structured polymorph of quartz), tuite (a HP polymorph of merrillite), wadsleyite (modified spinel-structured polymorph of olivine), and xieite (orthorhombic HP polymorph of chromite)^6,7,9,15,17–20^. A summary of the HP minerals, generally the most distinctive and unambigious evidence of strong shock events in L-chondrites, is given in Tomioka and Miyahara (2017)^21^.

The French historical fall Château-Renard is a highly shocked (shock stage S5) L6 chondrite^22^. Its initial mass of ~30 kg fell in 1841 and was recovered shortly (two days) after its fall. Château-Renard was an important highly shocked type specimen in early studies of the petrography, shock metamorphism, and impact geochronology of meteorites^18,23,24^. However, no verifiable occurrence of any HP mineral in this meteorite has been reported in the literature.

Here, we report new textural, compositional, diffraction, and micro-Raman spectroscopy data documenting HP minerals in this historical meteorite. From the HP minerals present and their textural association with melt veins and pockets, we infer constraints on the pressure-temperature conditions experienced during melt vein and melt-pocket formation in this meteorite. We further attempt to provide an estimate of the physical conditions of impact metamorphism due to a large collision event, commonly considered to be a single event experienced by the (single) L-chondrite parent body at ca. 470 Ma ago^25–27^. Finally, we obtain multiple compositional, spectroscopic, and structural constraints on a high-pressure pyroxene phase in Château-Renard and note the curious observation that this phase displays the Raman bands commonly associated with jadeite.

## Results

### General petrography of groundmass and melt veins

**Figure 1:**
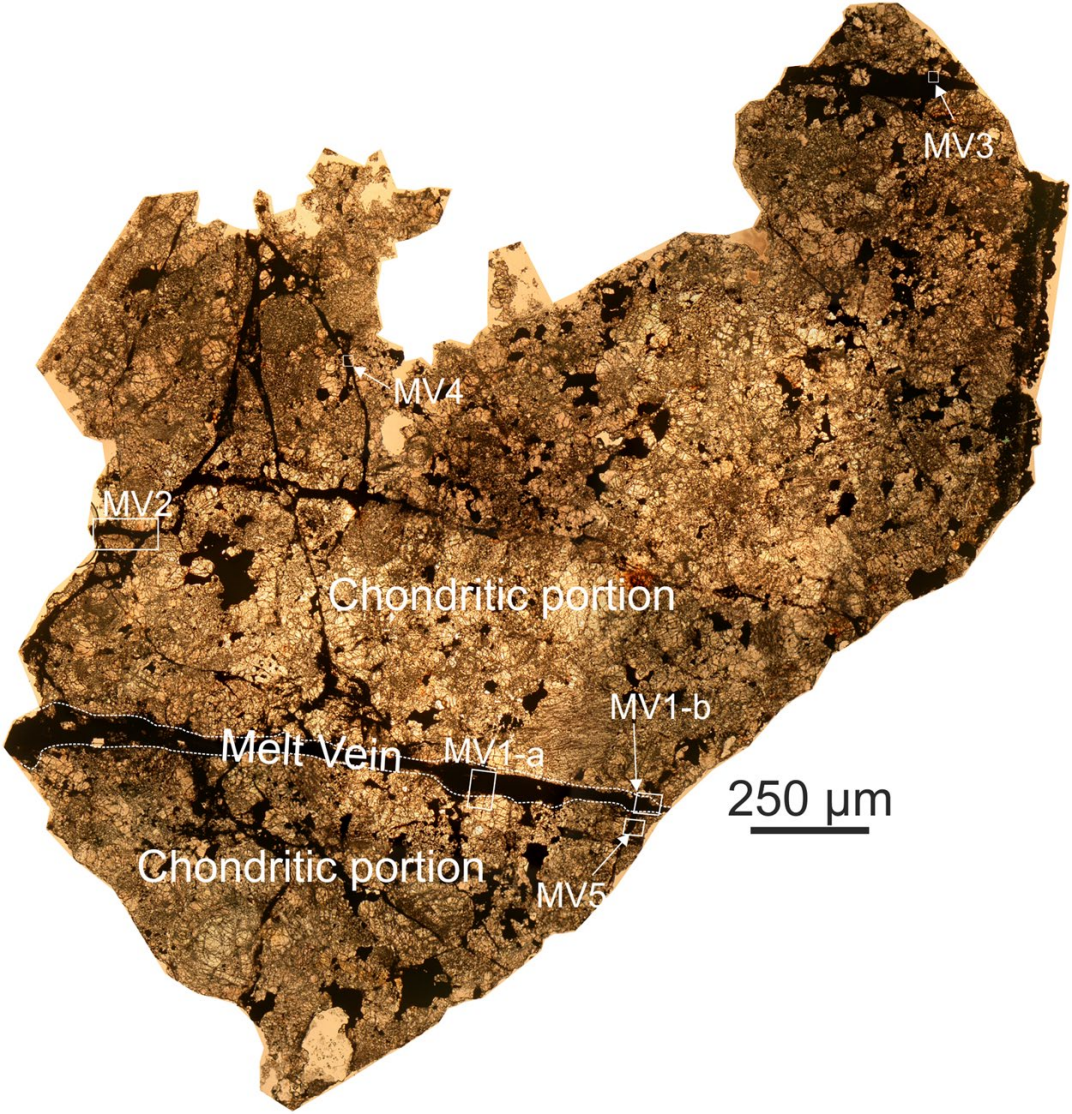
Château-Renard. Transmitted light mosaic image of Château-Renard polished section (L4361) showing the complex network of melt veins. Focus areas indicated by MV1-a, MV1-b, MV2, MV3, MV4 and MV5 are called out and enlarged in subsequent panels.

**Figure 2:**
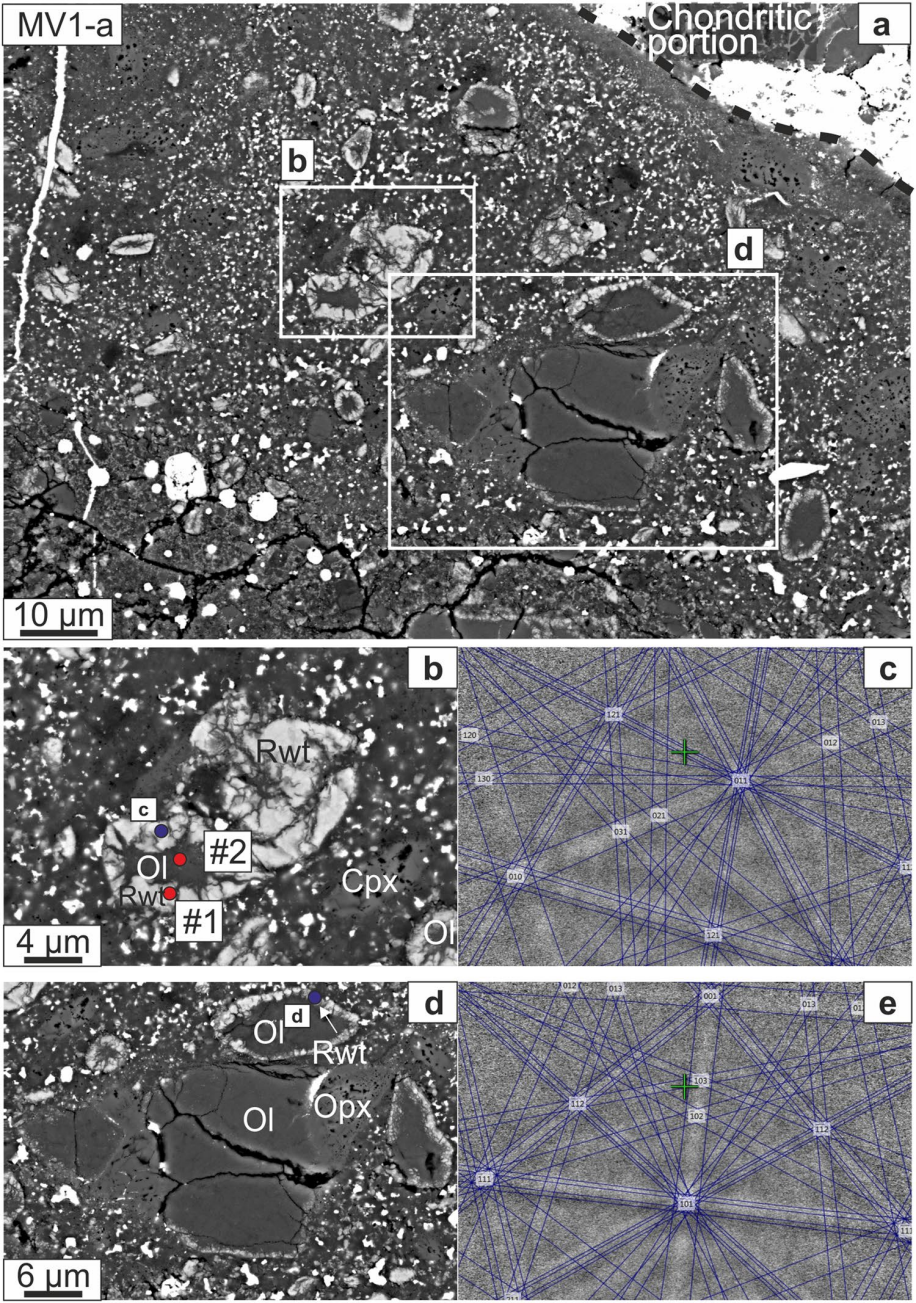
(**a**) BSE image of area MV1-a, where the main melt vein is ~100 μm wide; the areas enlarged in panel b and d are marked. (**b**) BSE image of a partially converted olivine grain; the bright rim shows the Raman bands of ringwoodite (Raman spectrum #1, Fig. 6a), whereas the partly enclosed dark area shows the Raman bands of olivine (Raman spectrum #2, Fig. 6a). (**c**) The EBSD pattern at point c on the bright rim indexed in the ringwoodite structure (Mean Angular Deviation (MAD) = 0.89°). (**d**) Another partly converted olivine grain with a backscatter-bright rim. (**e**) The EBSD pattern taken at point d also indexes as ringwoodite (MAD = 0.89°). The Raman and EBSD analysis positions are shown in this and subsequent figures as red and blue circles, respectively. Mineral abbreviations: Ol: olivine; Rwt: ringwoodite; Opx: orthopyroxene; Cpx: clinopyroxene.

**Figure 3:**
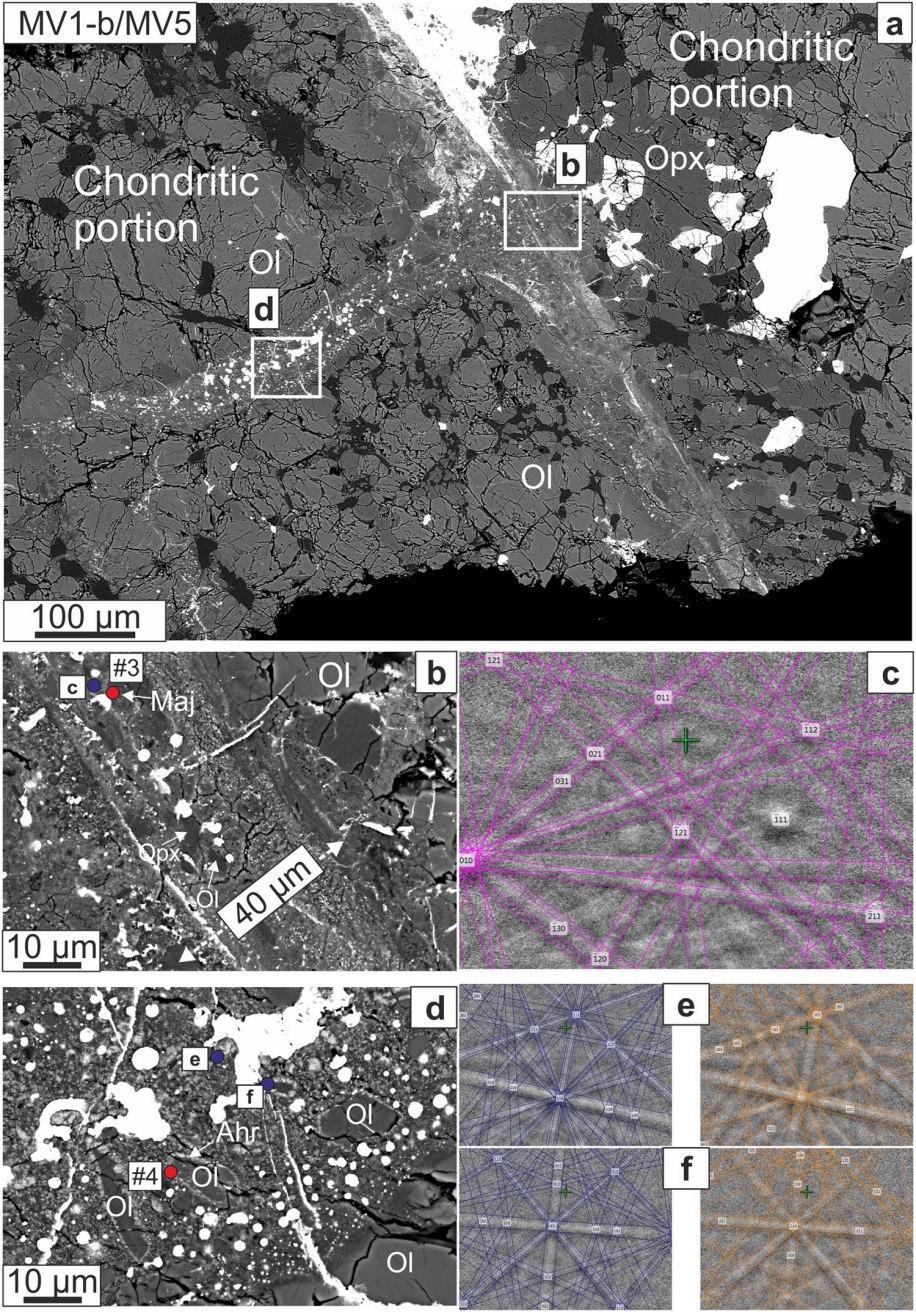
(**a**) BSE image of the intersection of MV5 with area MV1-a of MV1; the areas enlarged in panels b and d are marked. (**b**) Magnified BSE image showing the acquisition point for Raman spectrum #3 (Fig. 6a), with characteristic Majorite garnet band, and EBSD pattern. (**c**) EBSD pattern indexed and matched with garnet structure (MAD = 0.74°). (**d**) Magnified BSE image of vein MV5, perpendicular to but not cross-cutting MV1, featuring olivine partially transformed to ahrensite, with locations of EBSD pattern acquisitions at e and f and Raman acquisition at #4 (Fig. 6a). (**e**,**f**) The respsective EBSD patterns are shown with attempted indexing both in ahrensite structure at left (blue lines) and wadsleyite structure at right (orange lines). The patterns are similar and EBSD along does not distinguish in this case (MAD = 0.70° for both structures). Mineral abbreviations: Maj: majorite; Ahr: ahrensite.

**Figure 4:**
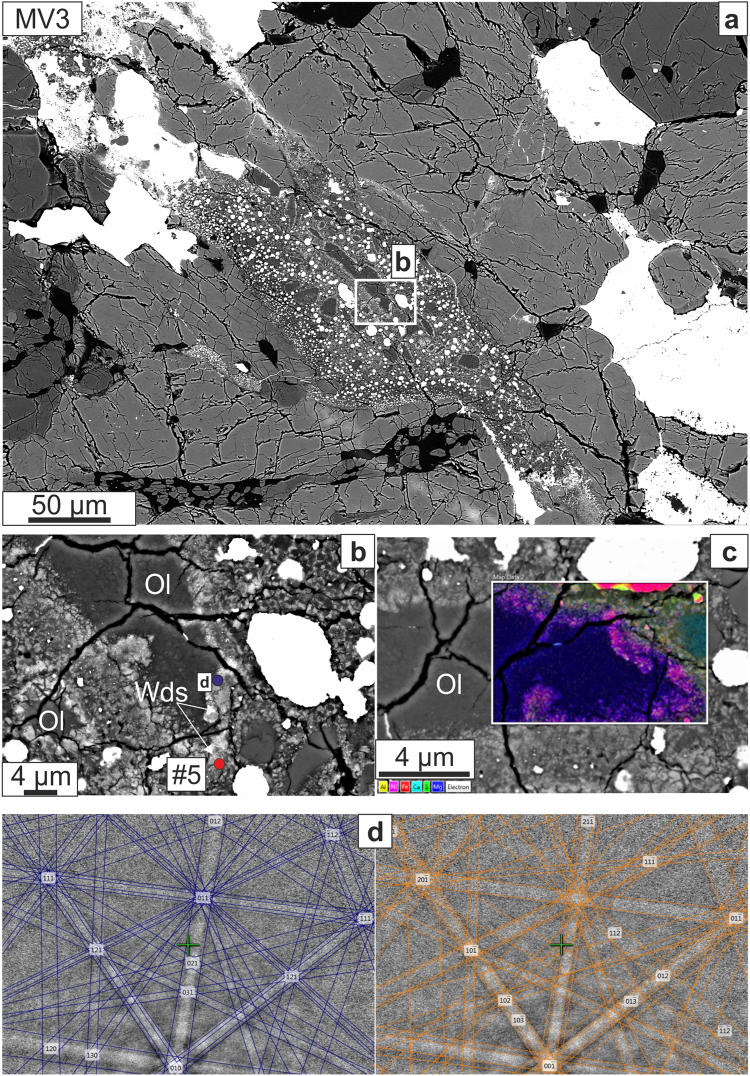
(**a**) BSE image of <100 μm wide vein (MV3); the areas enlarged in panel b and d are marked. (**b**) Partially converted olivine grains with Fe-enrichment towards the rims shown by increased backscatter contrast; the locations of EBSD acquisition at d and Raman acquisition at #5 (Fig. 6b) are marked. (**c**) Compound X-ray map for Al, Ni, Fe, Ca, S, and Mg with color scheme indicated; note different orientation than b. (**d**) The EBSD pattern from the rim of the converted olivine grains indexed as ringwoodite (left, blue pattern) and as wadsleyite (right, orange pattern). Although the patterns are similar, the pattern of allowed and forbidden double-reflections visible in the widths of the bands and the superposed (or not) bands is notably more consistent with the wadsleyite pattern (MAD = 0.72°), as expected from the Raman analysis at this location. Mineral abbreviations: Wds: wadsleyite.

**Figure 5:**
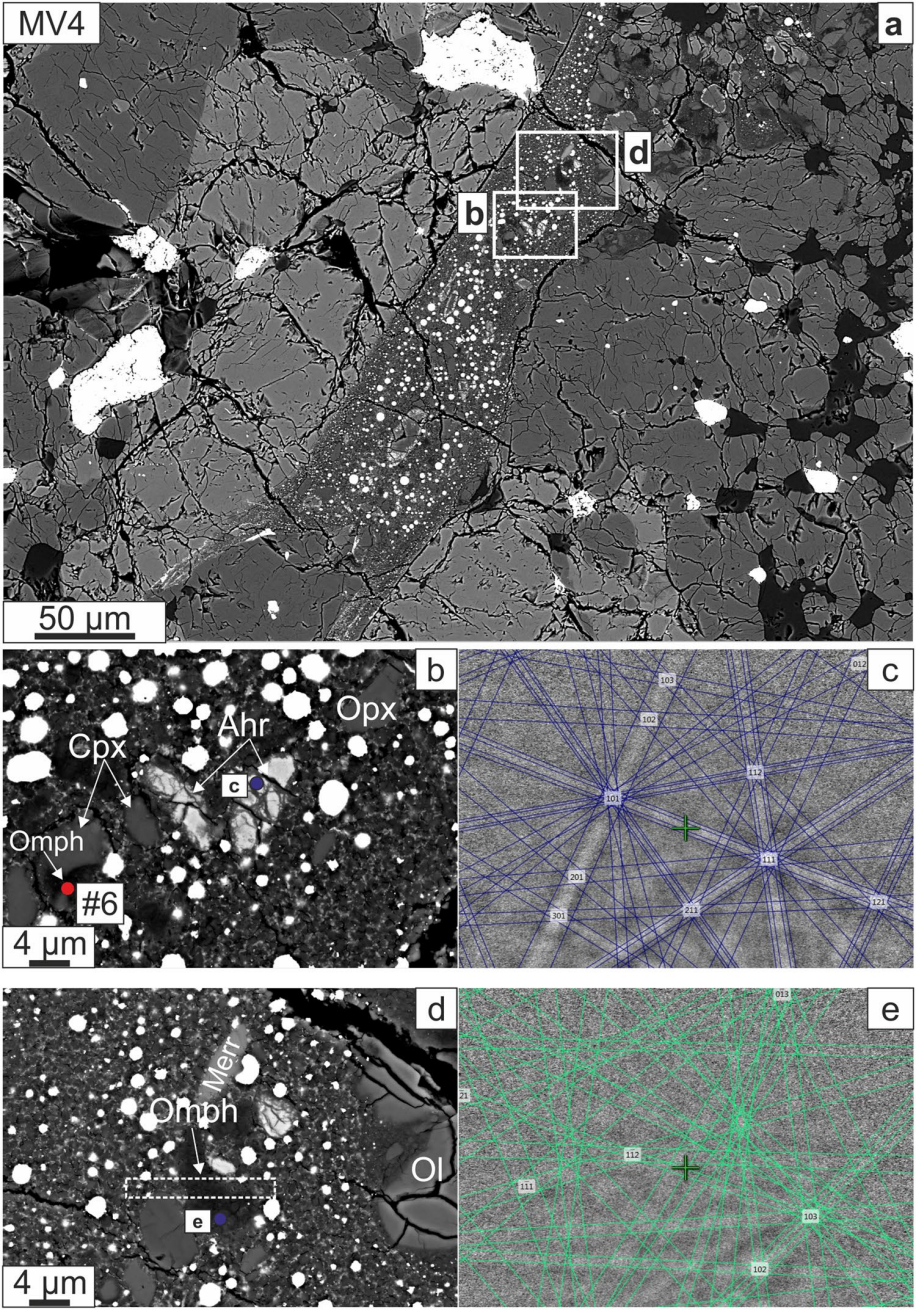
(**a**) BSE image of segment of MV4, with the areas enlarged in panels F1 and F3 marked. (**b**) Olivine fully transformed to ahrensite, showing location of EBSD pattern at c and Raman spectrum at #6 that shows jadeite peaks (Fig. 6c). (**c**) EBSD pattern indexed as ahrensite; EPMA analysis at the same point confirms ahrensite rather than ringwoodite (MAD = 0.71°). (**d**) BSE image of a Si-rich melt pool with jadeite composition showing location of EBSD pattern e and a white dashed rectangle indicating the area from which a FIB section was extracted for TEM analysis (see Fig. 7). (**e**) The EBSD pattern is indexed here as C2/c clinopyroxene but it is consistent with several different pyroxene polymorphs (MAD = 0.69°). Mineral abbreviations: Merr: merrilite; Opmh: omphacite.

In the groundmass of Château-Renard, olivine grains show strong mosaicism and planar fractures, and plagioclase is converted to feldspathic glass. There are numerous pervasive veins presumed to be the result of shock (Fig. 1). The MVs have variable thickness (from ~40 to ~200 μm; Fig. 1), are mostly crystalline (even at the nm scale) and are made up of silicate clasts (mostly of olivine stoichiometry), sulfides, and Fe-Ni metal grains (Figs 2–5). The MVs show characteristic gradation from glass-bearing rims, to segregated metal-rich layers ~20 μm from the MV boundary, to silicate clast-rich cores. Most clasts within the veins show shape preferred orientation with their long axis parallel to the MV elongation; where visible in backscattered electron (BSE) images of each vein, this will be pointed out below. We have analysed several of the silicate clasts in six selected regions of interest in melt veins found within a single polished thin-section of Château-Renard (NMHV-L4361; Figs 2–5 and Supplementary Figs 1–5).

### Characterization of HP minerals

Although no HP minerals have been previously reported in the Château-Renard meteorite, we have observed within the melt veins a number of HP minerals and report on them here for the first time, including ringwoodite, ahrensite, wadsleyite, and majorite. Within clasts and grains displaying olivine stoichiometry, there are clear correlations between the Fe/(Mg + Fe) ratio (expressed here as mole percent of the Fe_2_SiO_4_ fayalite component, Fa) and the occurrence of HP structures. In areas displaying the spinel structure, we place the formal divide between ringwoodite (the Mg_2_SiO_4_ endmember) and ahrensite (the Fe_2_SiO_4_ endmember) at Fa_50_. We also observe a sodic pyroxene that shares some features with jadeite; we document below our investigation of the identity of this mineral and show that it is not, in fact, jadeite.

We divide our observations according to the regions of interest that were studied, and in which we find the following assemblages of HP phases: (1) region MV1-a, a portion of the large vein MV1, contains ringwoodite + ahrensite (Fig. 2); (2) region MV1-b contains ringwoodite + wadsleyite + majorite (Fig. 3); (3) veins MV3 and MV5 both feature ahrensite + wadsleyite (Figs 3,4); and (4) vein MV4 contains sodic pyroxene + ahrensite + wadsleyite + clinoenstatite (Fig. 5). We note that the section also contains melt veins such as MV2 where no HP minerals were found at SEM scale (Supplementary Fig. 3).

#### MV1-a

**Figure 6:**
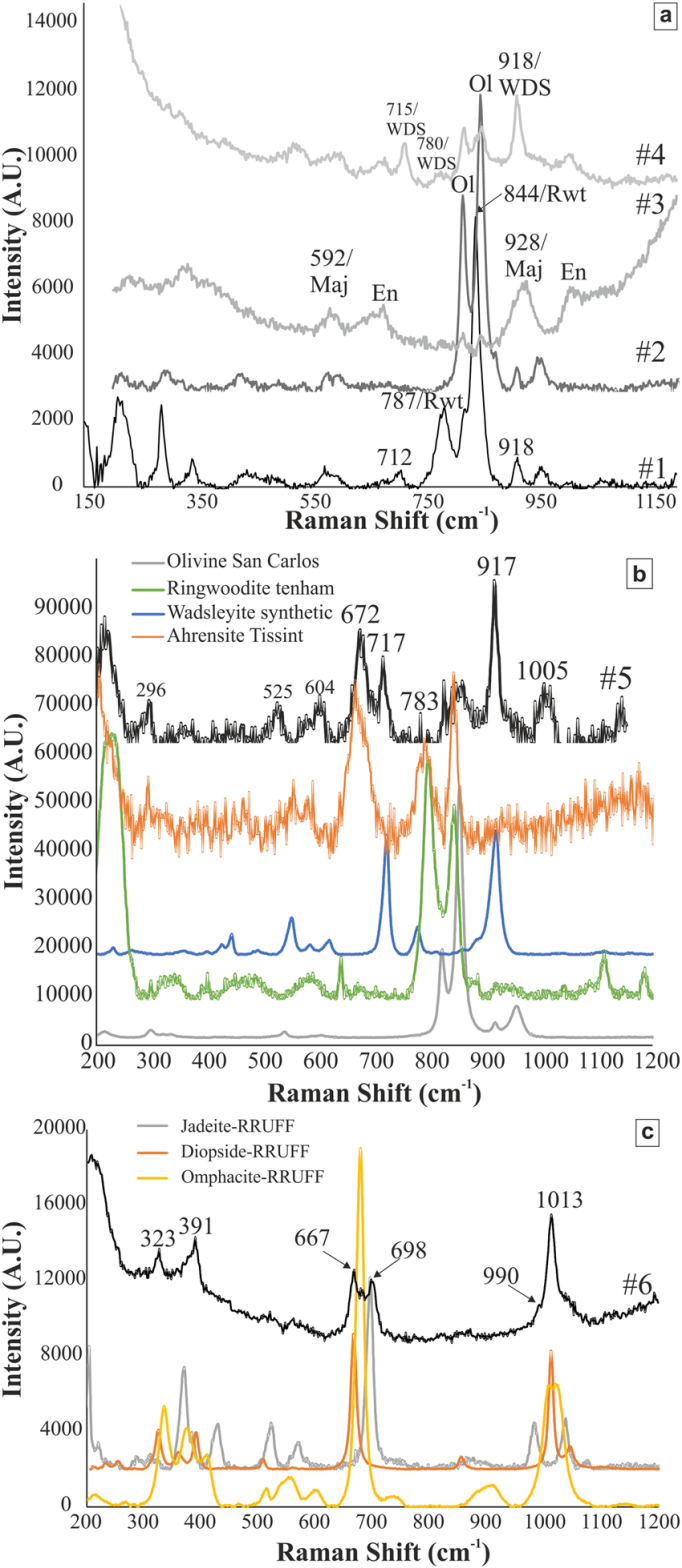
MicroRaman spectra for Château-Renard. (**a**) Spectrum #1 with peaks at 787 and 844 cm^−1^ is typical of ringwoodite. For comparison, the olivine spectrum (#2) from the core of the same grain (Fig. 2b) is also given. Spectrum #3 with enstatite doublet peak at ~662 and 680 cm^−1^, and ~1016 cm^−1^ are typical for orthopyroxene (Fig. 3b). In the same spectrum,, the peaks at 928 cm^−1^ and 592 cm^−1^ corresponds to majorite. Spectrum #4, with the intense peaks at ~715 and 920 cm^−1^, and less intense peak at ~780 cm^−1^ corresponds to wadsleyite (Fig. 3d). (**b**) Spectrum #5 shares the intense peaks of wadsleyite. RS spectra for olivine (San Carlos), ringwoodite (Tenham), wadsleyite (synthetic), and ahrensite (Tissint)^31^ are given for comparison. (**c**) Spectrum #6, with the characteristic jadeite peak at ~698 cm^−1^, and the minor peak as a shoulder at ~990 cm^−1^ obtained from the Si-rich pool given in Fig. 5b. Raman spectra for jadeite (#R070117), diopside (X050059), and omphacite (#R061129) from RRUFF database are given for comparison.

The zoned grains with olivine stoichiometry generally show, in BSE images, dark cores surrounded by bright rims. The average diameter of the entire grains is ~15 μm in width, while the rims are typically about ~5 μm wide. The Raman spectra collected from the bright rim regions display the characteristic bands at 784–789 cm^−1^ and 840–846 cm^−1^ of ringwoodite (Fig. 6a; spectrum #1) associated with the internal vibrations of the SiO_4_ tetrahedra^28^ (Raman modes T_2g_ and A_1g_). The dark regions display typical Raman spectra (Fig. 6a; spectrum #2) of olivine with all four of the major bands seen in the reference spectrum of San Carlos olivine (Fig. 6b). Turning to electron backscatter diffraction (EBSD) analysis coupled with high-spatial resolution co-located electron microprobe analyses (EPMA), the dark cores of the same grains display olivine structure with about 20 mol% Fe_2_SiO_4_ component (hence, the mineral species is forsterite; Supplementary Table 1). The bright rims are readily indexed with the spinel structure (space group *Fd*-3*m*; Fig. 2c,e). The majority of the bright spinel-structured rim areas have about 38–44 mol% Fe_2_SiO_4_ component and are classified as ringwoodite. However, the very bright rim surrounding the olivine in Fig. 2c is 67–69 mol% Fe_2_SiO_4_, well into the ahrensite compositional range (Supplementary Table 1). The point labeled ‘d’ (in Fig. 2d) on the bright rim of an olivine grain yields a spinel-structured EBSD pattern and ~50 mol% Fe_2_SiO_4_; this point is ambiguous in terms of mineral species assignment.

#### MV1-b

Raman spectrum *#*3, obtained from a grain located within this area (grain size ~18 μm in diameter with backscatter-bright rim ~4 μm wide), displays the characteristic major Raman peak at 928 cm^−1^ and minor peak at ~592 cm^−1^ (Fig. 6a) reported from both synthetic and natural majorite^8,29,30^. The major Raman peak of garnet is ascribed to the stretching of SiO_4_ tetrahedra^31^ (Raman mode A_1g_). The EBSD pattern collected from the same point can be indexed only with the garnet structure (Fig. 3c). The compositions of grains in this area (measured by EPMA) with these Raman and EBSD characteristics include points with essentially end-member orthopyroxene stoichiometry with 3.96 to 4 Si atoms per 12-oxygen formula unit (apfu) and very low (0.06 apfu) Al_2_O_3_ (Supplementary Table 1). Other points lie close to the majorite-pyrope join, with 3.58–3.62 apfu Si, Al_2_O_3_ up to 5.3 wt%, and CaO up to 3.4 wt%. The nearly end-member majorite composition has a formula Ca_0.14_Mg_2.98_Fe_0.84_Mn_0.06_Al_0.06_Si_3.96_O_12_, whereas the most Al-rich solid solution observed is Na_0.1_Ca_0.26_Mg_2.28_Fe_1.08_Mn_0.08_Al_0.46_Si_3.58_O_12_ and we label this phase majorite-pyrope_ss_ (Supplementary Table 1).

#### MV3 and MV5

The shape preferred orientation of large clasts of olivine with bright rims is quite apparent in Figs 3a and 4a. The bright rims of olivine (in MV3, grains are up to ~50 μm in longest dimension with rims ~4 μm wide; in MV5 grains are up to ~20 μm in longest dimension with ~3 μm rims) in these two veins show Raman spectra that appear to be combinations of ahrensite and wadsleyite (Fig. 6a,b). Although ahrensite and ringwoodite are isostructural and have several bands in common, spectrum #5 from MV5 (Fig. 6b) has additional bands at ~780 and ~670 cm^−1^ that are close to those in the ahrensite from the Tissint Martian meteorite^32^ (Fig. 6b). However, the same spectrum also shows bands at 714–718 cm^−1^ and 917–920 cm^−1^ similar to those seen in synthetic wadsleyite^33^ and assigned to Si_2_O_7_ symmetric stretch and SiO_3_ symmetric stretch, respectively^34^. The EBSD pattern of the thin bright rims in MV5 are indexed with spinel structure and confirmed to be ahrensite (~60 mol% Fe_2_SiO_4_) by high-resolution EPMA analysis (Supplementary Table 1). The Raman spectrum from point #4 in the MV3 region (Fig. 6a) is a relatively pure match to wadsleyite, including a minor peak at 780 cm^−1^. The wadsleyite spectra occasionally show olivine peaks at ~820 cm^−1^ and 852 cm^−1^, possibly indicating either incomplete transformation or partial back-transformation. The composition of the wadsleyite ri5 in MV3 (Supplementary Table 1) is Fa_41_, which is quite Fe-rich for wadsleyite; it is likely that this analysis includes unresolved areas of less Fe-rich wadsleyite and more Fe-rich olivine.

#### MV4

The distinctive sodic pyroxene in this area of Château-Renard displays the peaks considered to be characteristic of jadeite at ~700 cm^−1^ (Raman mode A_g_), ~990 (Raman mode A_g_), and ~1037 cm^−1^ (Raman mode A_g_) (Fig. 6c), as reported from simulated and experimental data on near-endmember jadeite^35^. We discuss in the next section of this paper whether these bands are in fact diagnostic of jadeite, what other pyroxene-structured phases might have very similar Raman spectra, and what additional observations are needed to confirm or reject the identification of jadeite.

In MV4, there are entirely bright and relatively homogeneous-looking fractured olivine-stoichiometry grains whose EBSD patterns suggest either wadsleyite or ahrensite (Fig. 5b,c), but the composition by SEM-EDS, ~66 mol% Fe_2_SiO_4_, is outside the stability field of wadsleyite^36^, so these points are most likely ahrensite as well. These may be rims of olivine grains whose cores are out of the plane of the section.

In MV4, in the area displaying Raman bands considered characteristic of jadeite, we find, by co-located EPMA and EBSD analysis, a pyroxene with formula in the range (Ca_0.07–0.10_Fe_0.16–0.19_Na_0.25–0.46_Mg_0.35–1.12_Al_0.29–0.57_)Si_2.01–2.04_O_6_ (Supplementary Table 1). We emphasize that it is quite unusual to be able to measure sodic pyroxene stoichiometry at EPMA scale in shock-melt veins of ordinary chondrites; most occurrences in the literature^8,11,37,38^ yield plagioclase stoichiometry due to intergrowth between sodic pyroxene and a Si-rich phase. Although this pyroxene is rich in the jadeite component (NaAlSi_2_O_6_), there are no points with Na formula units above 0.8. Hence, using the IMA nomenclature^39^, this is not jadeite but rather omphacite on the basis of composition. The EBSD pattern quality obtained on this material (Fig. 5e) is not adequate to distinguish between the space groups of jadeite (*C2/c*) and omphacite (*P2/n*), so this question was further studied by transmission electron microscopy (TEM). Although the high accelerating potentials used during TEM may cause substantial beam damage and reduce the Na count in TEM-EDS analysis, we stress that the compositional assignment to omphacite is here based on EPMA analysis, using analytical conditions under which sodic pyroxenes are typically beam-stable.

**Figure 7:**
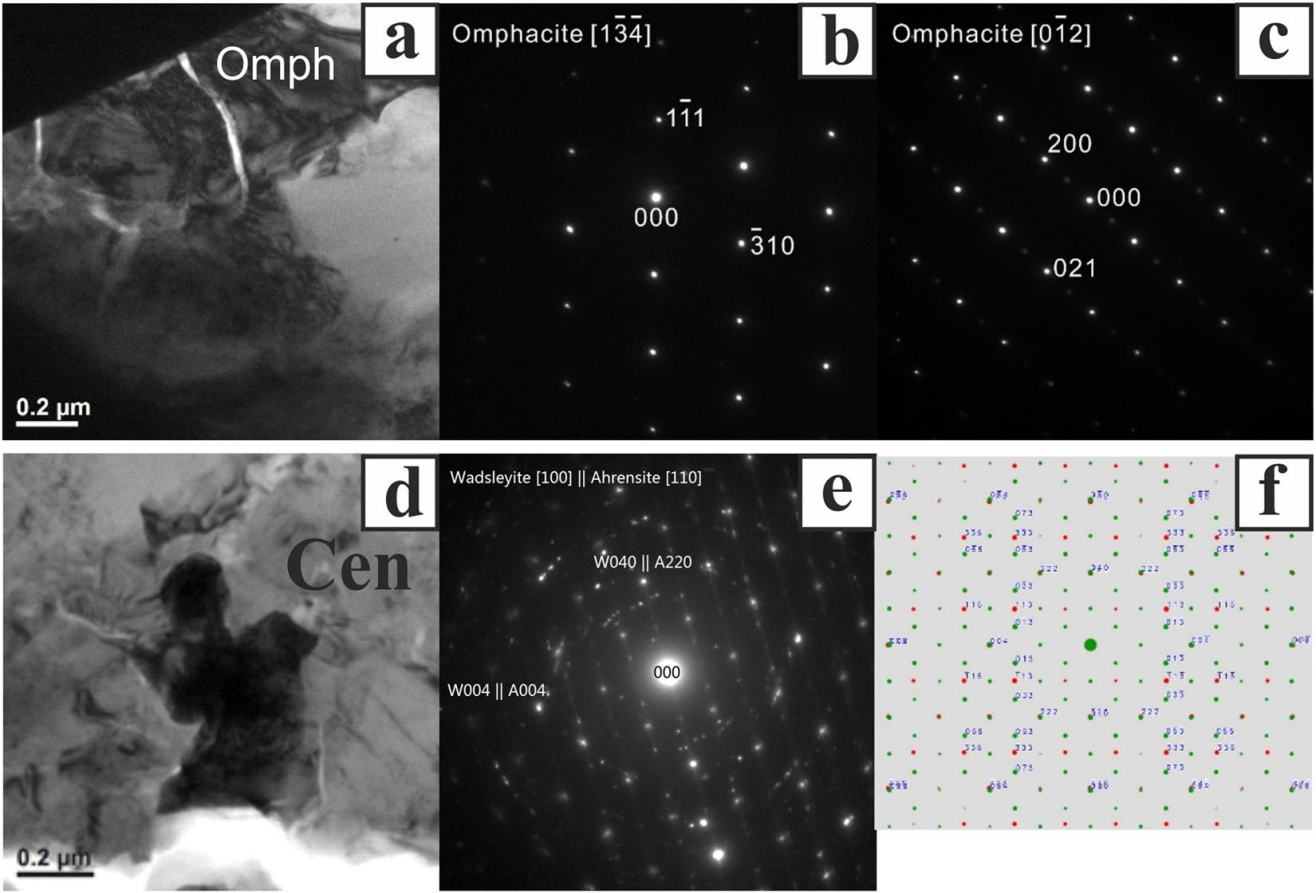
TEM results. (**a**) Bright field (BF) image of the omphacite (dark-grey contrast). SAED patterns of omphacite along the (**b**) [$1\overline{34}$] and (**c**) [012] zone axes. (**d**) BF image of intergrown ahrensite and wadselyite (dark contrast). The striation in the grain is inferred to be due to stacking faults. The surroundings are clinoenstatite (Cen). (**e**) SAED pattern of ahrensite [110] zone overlaps with wadsleyite [010] zone. (**f**) Simulated pattern showing the same topotaxis of ahrensite (red spots) and wadsleyite (green spots).

Focused ion beam (FIB) milling was used to extract an electron-transparent foil sampling the sodic pyroxene in area MV4 (Fig. 5a,d), allowing TEM analysis of the crystal structure by selected area electron diffraction (SAED). The foil reveals a Si-rich pool consisting of a clinopyroxene and Si-rich glass (Fig. 7). SAED patterns of the pyroxene are consistent with omphacite (*P*2/*n*) and plainly inconsistent with jadeite (*C2/c*). Although the structure and diffraction pattern of omphacite (*P*2/*n*) is very similar to low-clinopyroxene (*P*2_1_/*c*), the intensity distribution of the diffracted spots is more consistent with omphacite. The *h*00 (where *h* is an odd integer) spots are space-group forbidden in omphacite and any scattering towards these spots is the result of double diffraction, which is inefficient and makes low intensity spots. The SAED patterns indeed display very low intensities for all the odd *h*00 diffraction spots (Fig. 7b,c). The crystal structure is consistent with the composition from EDS analyses that show only 25–46% jadeite component in the sodic pyroxene.

In the TEM foil, we also observed ahrensite and wadsleyite (the spinel-structured polymorph is presumed to be ahrensite based on the observation of ~66 mol% Fe_2_SiO_4_ by SEM-EDS analysis that does not separarely resolve the two polymorphs and the typical partitioning of Fe between wadsleyite and the ringwoodite-ahrensite series; Supplementary Table 1) as intergrown grains crystallized from the melt (Fig. 7d). The two phases have a consistent topotaxial relationship, with the [100] zone-axis of wadsleyite parallel to ahrensite [110], as indicated by the overlapping diffraction patterns (Fig. 7d–f). Diffraction patterns also show streaks on ahrensite (110) and wadsleyite (010), suggesting stacking faults on these planes. Clinoenstatite (Fig. 7d) is the other silicate phase that crystallizes from the shock melt. Its composition (En_83_Fs_14_) is distinct from the sodic pyroxene in the Si-rich pool. From these observations we conclude that the mineralogy of the groundmass is fully consistent with the high-pressure minerals found as large grains. Hence, the groundmass material, likely to have crystallized from the melt at near-equilibrium conditions, leads to the same inferences about pressure and temperature conditions as the coarse grains despite the possibility of kinetic limitations on the achievement of equilibrium in the coarse grains.

## Discussion

### P-T-t constraints

This study presents the finding of four series of HP minerals (ringwoodite-ahrensite, wadsleyite, majorite-pyrope_ss_ garnet, and sodic pyroxene) in Château-Renard; all these HP minerals are described from this meteorite for the first time. Although we do observe an usual pyroxene coexisting with Na- and Si-rich melt, it is not jadeite either by composition or by structure. Omphacite is characteristic of eclogite-facies metamorphism and is probably an indicator of elevated pressure, but any pressure constraints based on experimental thresholds for the formation of jadeite *sensu stricto* are not relevant. Here we discuss the use of these observations to constrain the peak pressure, pressure-temperature evolution, and shock duration experienced by this meteorite.

**Figure 8:**
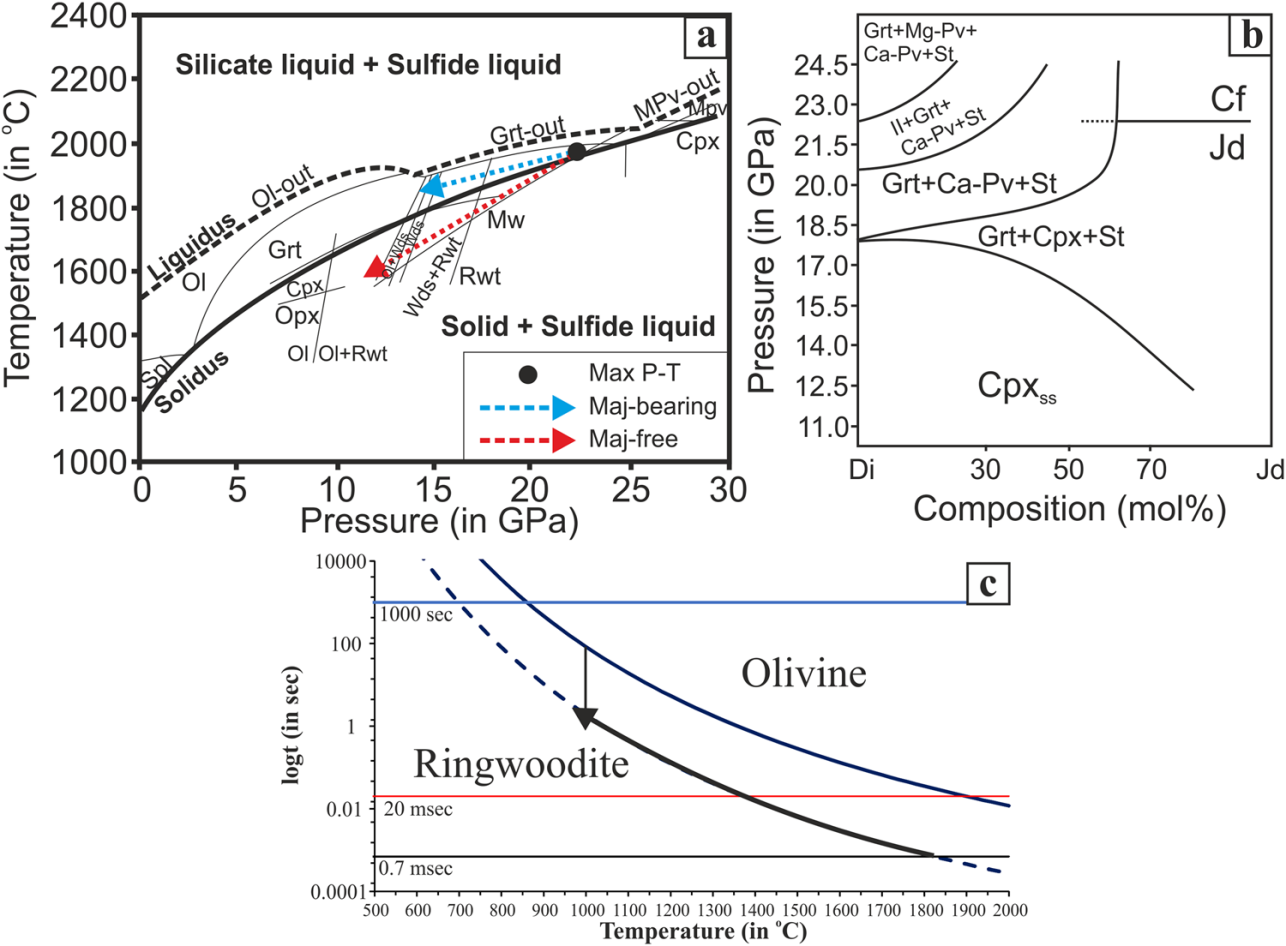
P-T (**a**,**b**) and Time-Temperature (**c**) diagrams. (**a**) The dotted arrows represent two possible P-T evolution paths through the majorite-bearing and majorite-free stability fields. Phase diagram boundaries are from Agee *et al*.^40^. (**b**) Pressure vs. composition phase diagram for the system Di-Jd (at 1600 °C)^46^. (**c**) Time-Temperature diagram for back-transformation of HP ringwoodite to LP olivine for the case of Château-Renard. The time for complete solidification of 0.7 msec suggests a maximum time remaining at high-T, while a shock pulse with duration of ca. 1 sec, is the maximum to avoid back-transformation of all ringwoodite to olivine, while at the same time the T should not exceed that of ca. 1000 °C.

If any part of the melt veins reached peak temperatures above the liquidus of the matrix material and maintained that state long enough to reach complete melting^38^, then it would have evolved according to the liquidus relations of a cooling chondritic liquid. At any pressure above the invariant point in the MgO-SiO_2_ system where the ringwoodite breakdown reaction intersects the liquidus, this sequence would begin with crystallization of (Mg,Fe)O periclase. Hence the absence of periclase + (devitrified) bridgmanite or periclase + stishovite suggests an upper bound for the peak pressure of ~23–25 GPa^40^. As shown in MV4, the matrix of MVs in Château-Renard crystalizes integrown wadsleyite-anhrensite plus clinoenstatite (Fig. 7). With the absence of garnet, this assemblage represents the solidus phase relation at 14–17 GPa (Fig. 8a). The solid-state transformations of the clasts in the MVs indicates consistent shock pressure, as discussed below.

In Château-Renard, we observe topotactic intergrowth of ringwoodite-ahrensite solid solution and wadsleyite from the shock melt. Many discussions of the shock pressures for olivine transformation have been based on an isochemical perspective reflecting the narrow transition intervals at a fixed terrestrial mantle-like composition close to Fa_12_. Viewed this way, peak pressures would need to have reached the stability range of ringwoodite, 17–23 GPa (Fig. 8a,b), whereas wadsleyite would imply a lower *P* range, 14–18 GPa, with only a small overlap. One explanation for their coexistence in the same meteorite or even in the same melt vein would be spatially or temporally variable pressure during the shock event, but we find the two phases intimately intergrown. Their coexistence might therefore be interpreted as a very specific constraint on pressure. However, this interpretation is not justified.

If the transformation took place from solid olivine precursors, then the transformation stress associated with the preferred solid-state transformation mechanism for the ringwoodite-wadsleyite transformation suggests a ~2 GPa range of coexistence^41^. We also note that the α-γ metastable reaction boundary lies in the middle of the wadsleyite stability field^42^ and transformation from olivine to ringwoodite exhibits a lower activation energy than olivine to wadsleyite. Ringwoodite may have nucleated first, in the stability field of wadsleyite, followed by topotactic growth of wadsleyite from ringwoodite nucleation sites.

A different approach considers the phase relations in the binary Mg_2_SiO_4_-Fe_2_SiO_4_ system, since the typical Fa content of L-chondrite olivines is nearly 25%. Compositional segregation during growth from a superliquidus state originally in the ringwoodite field would not explain the intergrowth: early crystallization of ringwoodite would enrich residual melt in Fe, moving it away from the stability field of wadsleyite. However, if a solid-state transformation occurred at high enough temperature to allow Fe-Mg interdiffusion, one could reconcile the observation of Fe-depleted forsterite olivine cores with moderately Fe-enriched wadsleyite, moderately Fe-enriched ringwoodite, and highly Fe-enriched wadsleyite-ahrensite intergrowths with a considerable range of pressures from 13–18 GPa (assuming equilibrium). The different HP polymorphs of olivine and the range of compsitions observed in the different melt veins could then be attributable to different cooling rates rather than to large variations in pressure.

Pressure indication from garnet depends significantly on the amount of Fe; the Château-Renard garnet has Fe/(Mg + Fe) in the range of 0.20–0.27, suggesting growth conditions of 17–20 GPa and 1800–2100 °C (Tomioka *et al*. 2016). The coexistence of majorite garnet (at the center of the MV) and wadsleyite (at the rim of the MV) indicates a thermal gradient present as the MV passed through this pressure range. Experimental observation shows that wadsleyite grains can grow at linear velocities up to <1 m s^−1^ and hence that the observed wadsleyite regions, 1–3 μm in size, require the MV to spend only a few microseconds in the wadsleyite stability field before quenching^43^.

Assuming that cooling and quenching of a MV is driven by thermal conduction across the boundary between the MV and its cooler matrix, we estimate a time for complete solidification of a typical Château-Renard MV of ~0.7 msec. The calculation assumes double-sided cooling of a 100 μm-wide slab of melt from super-liquidus temperatures (~2000 °C) while surrounded by cool matrix (~100 °C). Note, if peak temperature were below the liquidus, the cooling time would be slightly shorter. If the duration of the shock pulse were substantially shorter than the cooling time of the MV, then we would expect complete back-transformation to low-pressure minerals^13^. Specifically, the preservation of ringwoodite with about 40 mol% Fa component at the center of the MV suggests cooling below ~1000 °C while the rock was still at P > 13 GPa^44,45^ in order to prevent complete back-transformation of ringwoodite to olivine (Fig. 8c).

Raman spectra from the Na-Si-rich melt suggests the occurrence of a jadeite-like pyroxene. However, analytical TEM confirms that the pyroxene has the compositon and structure of omphacite. Relevant published phase diagrams^46,47^ show that addition of 50 mol% Di to jadeite lowers the low-*P* limit for a homogeneous cpx phase by about 0.5 GPa (from ~3 to ~2.5) and also lowers the upper-*P* limit for homogeneous cpx by about 5 GPa (from 21 GPa for the reaction Jadeite → Ca-ferrite to ~16 GPa for the reaction Clinopyroxene → Majorite + Ca-perovskite). Although preservation of the highest-*P* indicator minerals might be problematic, the presence of sodic clinopyroxene and the absence of Ca-ferrite, Ca-perovskite, or Ca-rich garnet suggests, at least locally, *P* ≤ 15.5 GPa in the pyroxene-bearing regions.

Given the diversity of mineral assemblages described within the single studied thin section, it is clear that Château-Renard records variable apparent pressure and temperature conditions. Possibly the different veins record different times along a common *P*-*T* path that they all experiened, depending largely on the local compositions, mineral kinetics, vein widths, and associated cooling rates. On the other hand, the presence of discrete veins directly proves heterogeneity of the temperature field, which is likely the result of collapse of spatially variable porosity during shock compression or slip along localized shear bands (despite some shape preferred orientation of large clasts parallel to the vein elongation, no convincing evidence of shear flow across the veins is observed). Shocking a heterogeneous medium also results in a heterogeneous pressure distribution. Although it is likely that pressure gradients on the order of GPa/mm would relax considerably after passage of the shock wave and before pressure release, it is hard to quantify the pressure differences that might persist over the potentially much shorter timescales involved in quenching the melt veins. Still, we lack a sound basis for asserting that a global peak *P*-*T* condition or global *P*-*T* path can be defined for the meteorite. Furthermore, as discussed below, the different veins may be recording altogether different shock events.

### Potential ambiguities of high-pressure pyroxene phase identification

Before attempting to use the presence of a HP mineral to document certain *P*-*T* conditions in a shock-metamorphosed object, such a phase must be thoroughly characterized and its phase identification confirmed by the combination of a structure-sensitive analytical method (such as Raman Spectroscopy, EBSD, XRD, and/− or TEM) and co-located compositional microanalysis (e.g., by EPMA). Jadeite, for example, is a crucial mineral reported in a number of L and H chondrites. The majority of these reports^8,30,37,38,48,49^ have combined Raman spectra with near-albite compositional analyses that do not resolve intergrowths of the pyroxene phase and silica. At least one published study has foregone compositional analysis and relied on Raman spectra alone^50^ and in another case the pyroxene in the Tissint meteorite later described and named as tissintite^51^ was misidentified as jadeite on the basis of its Raman spectrum despite compositional analyses indicating 62–66 mol % anorthite component^52^. Although the omphacite we have discovered could not explain the near-albite compositions reported for most jadeite occurences in L6 chondrites, our data do reveal that other sodic pyroxenes, with stability fields different from those of pure jadeite, may present Raman bands indistinguishable from those of jadeite. A compositional analysis is clearly required to confirm a Raman identification of jadeite before the stability field of jadeite can be used as a pressure minimum for a meteorite.

### HP mineral transformation mechanism; implications for duration of the shock pulse

Jadeite is commonly observed in ordinary chondrites as fine intergrowths of jadeite and silica with the bulk composition of albite^8,11,37,38^. Such jadeite presumably forms by solid-state decomposition of albite. On the other hand, the omphacite that we find in melt veins in Château-Renard combines components derived from more than one precursor phase — Na from plagioclase and Ca, Mg, and Fe from clinopyroxene. Hence is seems necessary that the omphacite grew from a melt whose formation digested both plagioclase and clinopyroxene. The melt then cooled enough to begin crystallizing while it remained at high enough pressure to stabilize omphacite.

HP polymorphs of olivine are found in two textural settings in the MV areas of Château-Renard. The TEM study of one MV shows ~10 nm-scale topotactic intergrowths of ahrensite and wadsleyite likely grown from shock melt. On the other hand, in several of the studied MV we find ringwoodite, ahrensite, or wadsleyite as incoherent μm-scale crystals collectively forming Fe-enriched rims enclosing large (5–50 μm) olivine grains. The latter texture, especially the presence of Fe-segregation towards wadsleyite or ringwoodite-ahrensite solid solutions, suggest a solid-state transformation mechanism with time for Fe-Mg interdiffusion. Similar textures were noted in L5 ordinary chondrite Dhofar 1970^53^ in a study that also emphasized that rapid cooling is required to prevent back-transformation to olivine.

We seek to quantify the necessary duration of the shock pulse by combining the Avrami equation (a general formalism for solid-state phase transformation) with an Arrhenius-type temperature dependence of the transformation rate constant. The result of this calculation (see Supplementary material) is summarized in Fig. 8. We estimate that, even if the veins were completely melted, they would cool to ~1000 °C within 0.7 ms and then cool much more slowly from this temperature (roughly the average of the peak vein temperature and the matrix temperature) over the ensuing several seconds. At this temperature, back reaction to olivine, if the pressure was released, would require about one second. We conclude that the pressure was maintained in the stability field of ringwoodite for at least one second, enabling continued cooling of the melt veins and preservation of ringwoodite. A pressure wave of this duration requires an impactor at least meters in scale.

### One single impact or many impacts?

The studied section contains a network of MVs of various widths that do, in some cases, intersect but do not reveal any crosscutting relations. Such a network of MVs might be simultaneously formed during a single impact event or might, even without evident cross-cutting, preserve evidence of a sequence of distinct impact events^54^. The question of whether this particular section of this particular L6 meteorite reflects one or multiple impacts may, in turn, bear on the overall impact history of all L-chondrites (if in fact they are derived from a single parent body) and, if so, on the question of how and when during the sequence of impacts that parent body was disrupted to yield the meteorites. Ar-Ar dating of Château-Renard shows a low-temperature Ar release ‘plateau’ of ~400 Ma^55^. It suggests that the rock was strongly shocked and degassed by an event much younger than the early Solar System formation ages of the chondrites and roughly consistent with L-chondrite parent body disruption at ~470 Ma^56^ (and references therein).

A range of contrasting shock conditions have been reported for various L6 chondrites. For example, Sahara 98222 clearly records much lower *P*-*T* conditions^11^ than Tenham^57,58^. Château-Renard, in turn, based on our observations, preserves in some veins peak *P*-*T* conditions intermediate between Sahara 98222 and Tenham (and distinct from both) and, in other veins (mostly thin), no HP minerals were observed (though they may be present at scales below our observations). The range in apparent peak *P* conditions within a single thin section is problematic for this discussion, unless attributed to differences in preservation. To the extent, though, that (1) a characteristic peak *P* can be defined for each meteorite, (2) these peak pressures differ among the meteorites, and (3) there was a single parent body, the single-impact scenario then implies that each meteorite was derived from a distinct location relative to the impact point, with shock pressure systematically decreasing with increasing distance from the point of impact. Alternatively, a single-parent body and multiple-impact scenario allows for a range of impact velocities and impactor densities and sizes as well as different locations in the target. Of course, in a multiple parent-body scenario, or one in which some of the impact events recorded by the shock metamorphic assemblage in some L-chondrites followed the disruption of an original single body, there need not be any relationship among the observations in different meteorites.

In this context, careful examination of the different MVs within Château-Renard for the presence of HP minerals is important. We have identified HP minerals in MVs over a considerable range of MV widths, from 50 to ~200 μm. Because preservation of HP minerals is thought to require thermal quench before the release of the high-pressure pulse, the thinnest melt veins (30–50 μm) provide the most direct constraint on peak *P* whereas the thickest melt veins that still have HP minerals provide the best bound on shock-pulse duration. Yet, the thinnest MV that we inspected at field-emission SEM scale (MV2, Supplementary Fig. 3) in our section of Château-Renard revelated no evidence of HP minerals. Of course, other differences among the MVs might offer alternative explanations for the differences in HP mineral formation or preservation; e.g., the thickest MV is enriched in silicate clasts whereas the thin MVs tend to be richer in sulfides (mainly troilite). Nevertheless, our inability to find HP minerals in a thin melt vein suggests that we should entertain the hypothesis that our section contains two generations of MVs representing different shock events. If so, we should also attempt to define which came first and how much time elapsed between these two events. No cross-cutting relationship is observed to prove either scenario. If we assume that the wide, silicate-rich, HP mineral -bearing veins formed after the narrow, sulfide-rich, possibly HP mineral-free veins, then the second event likely reached higher peak pressure, sufficient to form the HP assemblage of majorite-pyrope_ss_ garnet + ringwoodite. As the first-generation MVs would have already been consolidated during the first impact, they would have lacked the porosity or focused shear deformation needed to generate local high temperature, melting, and HP mineral growth during the second impact. A critical challenge for a multiple-impact scenario, though, is the persistence through the first impact event of porosity sufficient to provide locations of focused heating leading to MV formation during the second impact.

A “two impact events in a very short time interval” hypothesis was already proposed for the Sixiangkou L6 ordinary chondrite^54^. However, thermal modeling using Finite Element Heat Transfer calculations from other L6 chondrites demonstrates that the time gap between the formation and solidification of MVs of similar thickness was at most a few seconds^14^. In that context, a possible resolution is a two-pass interaction in which an impactor fragmented during a close approach to the parent body; the fragments continued to travel on related orbits and each impacted sequentially on the next close approach. This scenario can yield multiple impact events on the parent body within a period of a few seconds.

## Conclusion

Château-Renard is a highly shocked L6 ordinary chondrite containg numerous HP minerals, including ringwoodite, ahrensite, wadsleyite, majorite, and sodic pyroxene. These HP minerals occur within thick melt veins (>50 μm in width), whereas the thinner MV that we inspected apparently lacks HP minerals. The HP assemblages are (1) ringwoodite + wadsleyite; (2) ringwoodite + wadsleyite + majorite-pyrope_ss_; (3) ahrensite + wadsleyite; and (4) sodic pyroxene + ahrensite + wadsleyite + clinoenstatite. The absence of periclase + (retrogressed) bridgmanite or periclase + stishovite suggests an upper bound for the peak pressure of ~23–25 GPa, whereas the presence of ringwoodite and majorite suggest peak pressures in the range of 17–23 GPa. Furthermore, co-occurrence of ringwoodite-ahrensite solutions with wadsleyite implies a modestly lower *P* range, 14–18 GPa (or less accounting for Fe-rich compositions), along with rate-controlled nucleation of the HP polymorphs of olivine. On the other hand, using binary jadeite-diopside phase diagrams to estimate the pressure implied by the occurrence of omphacitic pyroxene suggests peak P ≤ 15.5 GPa. The inconsistency of ≥1.5 GPa in these pressure estimates suggests that either spatial heterogeneity, temporal evolution, multiple impact events, or some combination of these are recorded by the various HP mineral assemblages in the investigated section. In addition, the temperatures estimated for majorite growth (≥1800 °C) in the MV centers and wadsleyite formation (≤1500 °C) at the MV edges require a temperature gradient during HP mineral growth. Without cross-cutting relations between the various melt veins, it is challenging to distinguish among the various explanations for preservation of heterogeneous conditions.

## Methods

### Sample Characterization

Eight historical covered thin sections (A981, J1855, J1856, J1857, J1858, J2890, J2891, and J2892) and one polished thick section (L4361) of Château-Renard from the collection of the Natural History Museum, Vienna (NHMV) were investigated. Melt veins were examined under the optical microscope, focusing mainly on clast-rich regions, and we then conducted a careful search for shock effects in mineral grains and HP minerals.

### Scanning Electron Microscopy and Electron Backscatter diffraction

The section was carbon-coated and investigated with the NHMV JEOL JSM-6610 LV equipped with a highly sensitive backscattered electron detector and an energy-dispersive X-ray spectrometer (EDS). Analyses were conducted using a 15 kV accelerating voltage and a ∼20 nA probe current, yielding analytical volumes with diameters less than 3 μm.

Additional SEM analyses were performed at the California Institute of Technology (Caltech) GPS using a Zeiss 1550VP field-emission scanning electron microscope equipped with an angle-sensitive backscattered electron detector, 80 mm^2^ active area Oxford X-Max Si-drift-detector EDS, and an HKL EBSD system. SEM imaging and EDS analyses used a 15 kV accelerating potential and a 120 μm field aperture in high-current mode (∼4 nA probe current), yielding imaging resolution better than 2 nm and an activation volume for EDS analysis ∼1–2 μm^3^ on silicates. Single crystal EBSD analyses at a sub-micrometer scale were performed at 20 kV and 6 nA in focused beam mode with a 70° tilted stage on uncoated specimens in “variable pressure” mode (25 Pa of N_2_ gas in the chamber to reduce specimen charging). Imaging, mapping, semi-quantitative EDS analysis, and EBSD were conducted using the SmartSEM, AZtec, and Channel 5 software packages.

### Electron Probe Microanalysis

Major element compositions of matrix and MV minerals were determined using a JEOL JXA8530F Field Emission EPMA instruments (FE-EPMA) equipped with five wavelength-dispersive spectrometers (WDS) and one energy-dispersive spectrometer (EDS) at both the NHMV and the Institut für Mineralogie, University of Münster, Germany. Mineral analyses were performed with an accelerating voltage of 15 kV. For minerals, a 20 nA focused beam current, 20 s counting time on peak position, and 10 s for each background were used. For glass analyses, a slightly defocused (5 μm diameter) beam, 5 nA probe current, and counting times of 10 s on-peak and 5 s on each background position were used. Natural mineral standards used were albite (Na, Si, Al), wollastonite (Ca), olivine (Mg), almandine (Fe), spessartine (Mn), orthoclase (K), rutile (Ti), chromite (Cr), and Ni-oxide (Ni) with ZAF matrix correction.

### Transmission Electron Microscopy

We used a FEI Nova 600 Nanolab DualBeam focused ion beam (FIB) and scanning electron microscope (SEM) for the sample preparation and lift-out. The sample thinning was finalized with an 8 kV, 19 nA Ga-ion beam. The analytical transmission electron microscopy (ATEM) analysis was performed on FEI Tecnai TF20 with super-twin objective lens, operated at 200 kV. The EDS data were collected in TEM mode using a EDAX SiLi detector with 10 eV/channel and 51.2 µs process time, to achieve 500 cps signal and 20–50% deadtime. The FIB and TEM facilities are in the Kavli Nanoscience Institute at Caltech.

### Micro-Raman Spectroscopy

Raman spectra for preliminary phase identification were conducted on the polished thin section using a dispersive confocal Raman microscope, Renishaw inVia Reflex at the National Hellenic Research Foundation. Analyses used a a 514 nm Ar-ion laser and a ×100 objective lens and spectra were collected in the Stokes region for Raman shifts from 200–1600 cm^−1^. Additional Raman analyses were performed at the Open University, Milton Keynes, United Kingdom, using a Horiba Jobin-Yvon LabRam HR Raman Microscope equipped with both 514 nm and 633 nm lasers. The laser beam was spread across ~1–2 μm spots at relative low incident power (ca. 5 mW) in order to avoid sample destruction. For each spot analysis on the Open University system, we averaged spectra over 5 consecutive 60 sec accumulation times. Gaussian-Lorentzian peak fitting (Spectragryph version 1.0.5) was used to remove background and estimate the peak centers. Collected spectra were compared with published data from RRUFF and the Handbook of Raman Spectra. The locations of each Raman spot analysis were recorded and co-located EPMA analytical points were collected in order to couple structural and compositional characterization at common spots.

### Modeling strategies

#### Time for complete solidification of Melt Veins

The time required for complete solidification of a melt inside a tabular vein was estimated following the procedures of Turcotte & Schubert and Langenhorst & Poirier)^59,60^. In this model, it is assumed that the melt vein is surrounded by totally solid material at temperature T_0_, while the interior of the vein is totally melted at temperature T_m_. A vein represented as a hot slab of a thickness 2*w* will cool and solidify in a characteristic time *t*_*s*_^60^

1 $${t}_{s}=\frac{{w}^{2}}{4\cdot \kappa \cdot {\lambda }^{2}}$$

where κ is the thermal diffusivity and λ is a dimensionless coefficient that accounts for the boundary conditions and latent heat. λ is obtained by substitution of the following two boundary conditions at the moving front

2 $$\frac{d{y}_{m}}{dt}=-\,\lambda \cdot {(\frac{\kappa }{t})}^{0.5}\,$$

3 $$\,{(\frac{\partial T}{\partial y})}_{y={y}_{m}}=\frac{-({T}_{m}-{T}_{0})}{{(\pi \kappa t)}^{0.5}}\cdot \frac{{e}^{-{\lambda }^{2}}}{(1+{\rm{erf}}[\lambda ])},$$

into the conservation equation

4 $$\rho \cdot L\cdot \frac{d{y}_{m}}{dt}=\kappa \cdot {(\frac{\partial T}{\partial y})}_{y={y}_{m}}$$

resulting in

5 $$\frac{L\cdot \sqrt{\pi }}{{C}_{P}\cdot ({T}_{m}-{T}_{0})}=\frac{{e}^{-{\lambda }^{2}}}{\lambda \cdot (1+{\rm{erf}}[\lambda ])},$$

where *L* is the latent heat of crystallization, *C*_*p*_ is the specific heat and *erf* is the error function. Also, when the vein solidifies, the temperature at the boundary with the surrounding matrix material will be constant and is given by

6 $${T}_{b}={T}_{0}+\frac{{T}_{m}-{T}_{0}}{1+{\rm{erf}}[\lambda ]}$$

The values we used for the modeling for Château-Renard case were: *L* = 320 kJ kg^−1^, *C*_*p*_ = 1.2 kJ K^−1^ kg^−1^, κ = 10^−6^ m^2^ sec^−1^, *T*_m_ = 2000 °C and *T*_0_ = 100 °C. These parameters yield λ = 0.93, *T*_b_ = 1148 °C, and 0.72 ms for a typical 100 μm width vein in Château-Renard.

#### Preservation of HP minerals

We estimated the time over which HP minerals might persist without back-transformation to their low-pressure equivalents. We combined the Avrami equation (which describes how solids transform from one phase to another at constant temperature) and an Arrhenius relationship for the transformation rate constant, resulting in

7 $$t\,={[-\frac{\mathrm{ln}(1-X)}{A\cdot exp(-\frac{E}{RT})}]}^{\frac{1}{n}}$$

where *X* is the volume fraction of the transformed phase, *A* is a frequency factor, *E* the activation energy for the polymorphic transformation, *R* the gas constant, *n* a constant determined by the dimensionality of the nucleation and growth processes (surface vs. volume), and *T* is absolute temperature. In our model we used the following numbers: *X* = 0.99, *A* = 2.44 × 10^11^ (from Arrhenius equation for Mg_1.6_Fe_0.4_SiO_4_), *E* = 324149.9 J/mol^61,62^, *R* = 8.3144598 J/K mol, n = 1.52 (value used by Sato *et al*.^63^), and *T* in the range given in Fig. 8c.

Assuming that the shock event that disrupted the L-chondrite parent body happened at 470 Ma, the preservation of ringwoodite, majorite and wadsleyite over this time requires that the temperature be maintained below the conversion boundary curves for this time period in *t*-*T* space. For both the olivine and pyroxene systems, the upper bound temperature corresponds to ca. 200–250 °C.

### Data Availability

The datasets generated during and/or analysed during the current study are included in this published article (and its Supplementary Information files) but also are available from the corresponding author on reasonable request.

## Electronic supplementary material

Supplementary information
