# Supplementary material for: High pressure minerals in the Château-Renard (L6) ordinary chondrite: implications for collisions on its parent body
Source: Sci Rep. 2018 Jun 29;8:9851. doi: 10.1038/s41598-018-28191-6 (PMC6026127; doi:10.1038/s41598-018-28191-6)
Supplement: Supplementary file 1 — Supplementary information [file 41598_2018_28191_MOESM1_ESM.docx]

**High pressure minerals in the Château-Renard (L6) ordinary chondrite: implications for collisions on its parent body**

Ioannis Baziotis^1^, Paul D. Asimow^2*^, Jinping Hu^2^, Ludovic Ferrière^3^, Chi Ma^2^, Ana Cernok^4^, Mahesh Anand^4,5^, Dan Topa^3^

^1^Department of Natural Resources Management and Agricultural Engineering, Agricultural Univ. of Athens, Iera Odos 75, 11855 Athens, Greece, ibaziotis@aua.gr,

^2^California Institute of Technology, Division of Geological and Planetary Sciences, Pasadena, California 91125, USA,

^3^Natural History Museum, Burgring 7, A-1010 Vienna, Austria,

^4^Planetary and Space Sciences, The Open University, Milton Keynes MK7 6AA, United Kingdom.

^5^Department of Earth Sciences, The Natural History Museum, London, SW7 5BD, United Kingdom.

*: Corresponding author (asimow@gps.caltech.edu)

| **Table 1: Major-element composition (wt%) of minerals observed in selected melt veins from *Château-Renard*** | | | | | | | | | | | | | | | | | | |
| --- | --- | --- | --- | --- | --- | --- | --- | --- | --- | --- | --- | --- | --- | --- | --- | --- | --- | --- |
|  | MV1-a | | | | MV1-b | | | MV2 | | | | | MV3 | MV4 | | | MV5 | |
|  | Ol | Rwt | Ahr | Rwt | | Maj | Maj-Prp_ss_ | | Ol dark core | Ol  bright rim | Opx  Al-poor | Opx  Al-  rich | Wds | Na-Cpx | Na-  Cpx | Ahr | Ahr | Wds |
| ** | EPMA | EPMA | EPMA | EPMA | | EPMA | EPMA | | EPMA | EPMA | EPMA | EPMA | EPMA | SEM | SEM | SEM | SEM | SEM |
| *** | 1 | 2 | 3 | 4 | | 5 | 6 | | 7 | 8 | 9 | 10 | 11 | 12 | 13 | 14 | 15 | 16 |
| SiO_2_ | 38.5 | 37.2 | 34.1 | 36.8 | | 55.5 | 49.0 | | 39.2 | 36.5 | 53.8 | 50.4 | 36.8 | 58.0 | 61.2 | 32.7 | 33.6 | 41.1 |
| TiO_2_ | nd^‡^ | 0.04 | 0.04 | nd | | 0.18 | 0.10 | | nd | nd | 0.16 | 0.14 | 0.03 | nd | nd | nd | nd | nd |
| Al_2_O_3_ | 0.09 | 1.04 | 0.31 | 0.55 | | 0.33 | 2.79 | | 0.02 | 1.36 | 0.64 | 5.7 | 0.74 | 14.1 | 17.5 | 0.8 | 1.2 | nd |
| Cr_2_O_3_ | 0.02 | 0.13 | 0.12 | 0.11 | | 0.23 | 0.33 | | 0.03 | 0.11 | 0.07 | 0.19 | 0.03 | nd | nd | nd | nd | nd |
| FeO | 18.65 | 37.2 | 48.6 | 35.3 | | 13.0 | 15.8 | | 14.2 | 41.9 | 13.9 | 16.1 | 33.4 | 6.5 | 4.3 | 51.3 | 47.8 | 14.4 |
| MnO | 0.26 | 0.12 | 0.08 | 0.17 | | 0.51 | 0.68 | | 0.37 | 0.15 | 0.52 | 0.32 | 0.65 | nd | nd | nd | nd | nd |
| MgO | 41.4 | 25.0 | 17.4 | 27.9 | | 29.3 | 27.9 | | 45.9 | 18.9 | 28.2 | 23.8 | 27.3 | 11.5 | 6.5 | 14.8 | 16.9 | 44.5 |
| CaO | 0.10 | 0.36 | 0.15 | 0.28 | | 1.07 | 2.12 | | 0.09 | 0.36 | 0.76 | 1.41 | 0.46 | 2.6 | 2.2 | 0.4 | 0.6 | nd |
| NiO | 0.07 | 0.05 | 0.05 | 0.08 | | 0.12 | 0.10 | | 0.06 | 0.07 | 0.08 | 0.07 | 0.1 | nd | nd | nd | nd | nd |
| P_2_O_5_ | nd | 0.07 | 0.07 | 0.03 | | 0.04 | 0.10 | | nd | 0.05 | nd | 0.06 | 0.01 | nd | nd | nd | nd | nd |
| Na_2_O | nd | nd | nd | nd | | 0.08 | 0.35 | | nd | nd | 0.22 | 2.42 | nd | 6.9 | 7.6 | nd | nd | nd |
| K_2_O | nd | nd | nd | nd | | nd | nd | | nd | nd | nd | nd | nd | 0.5 | 0.7 | nd | nd | nd |
| Total | 99.1 | 101.2 | 100.9 | 101.3 | | 100.4 | 99.2 | | 99.9 | 99.4 | 98.4 | 100.5 | 99.4 | ** | ** | ** | ** | ** |
| Fe#* | 20.2 | 45.5 | 61.1 | 30.8 | | 49 | 53 | | 33.1 | 61.1 | 47.9 | 40.4 | 40.7 | 24.4 | 26.7 | 66.1 | 61.1 | 15.4 |
| ‡: nd = not detected; EPMA detection limit ~0.01 wt. %; SEM detection limit ~0.1 wt. %  *: Fe# = FeO/(FeO+MgO)·100; for olivine polymorphs this is equivalent to mol. % fayalite component  **: EPMA = Electron Microprobe WDS; SEM = Scanning Electron Microscope EDS. SEM analyses are normalized to 100% total. See methods section for analytical protocols.  ***: The number corresponds to the analysis location in the supplementary figures 1-6. | | | | | | | | | | | | | | | | | | |


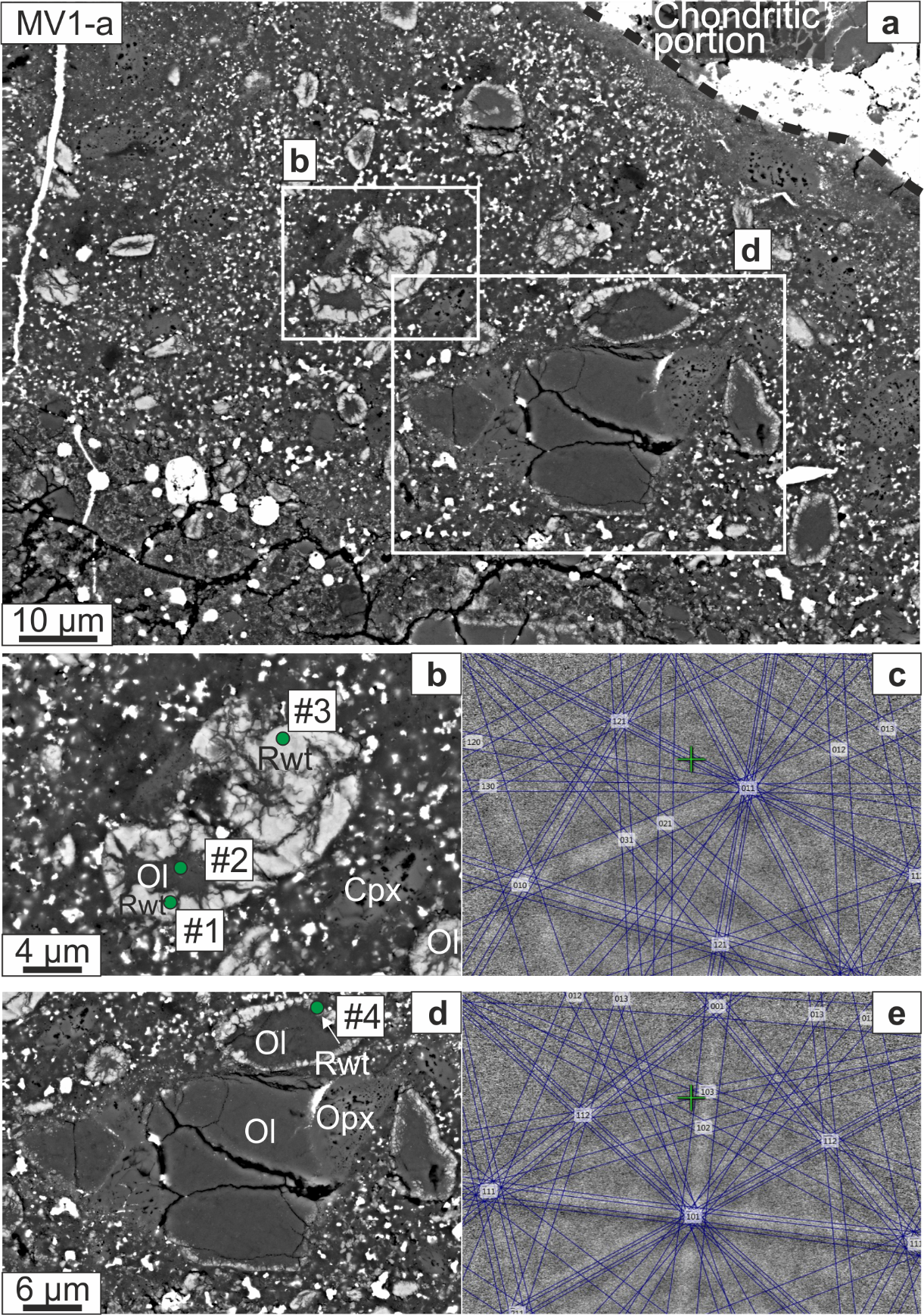


**Supplementary Figure 1:** *Same as in figure 2 annotated for EPMA spots (green coloured circles). Mineral abbreviations: Ol: olivine; Rwt: ringwoodite; Opx: orthopyroxene; Cpx: clinopyroxene.*


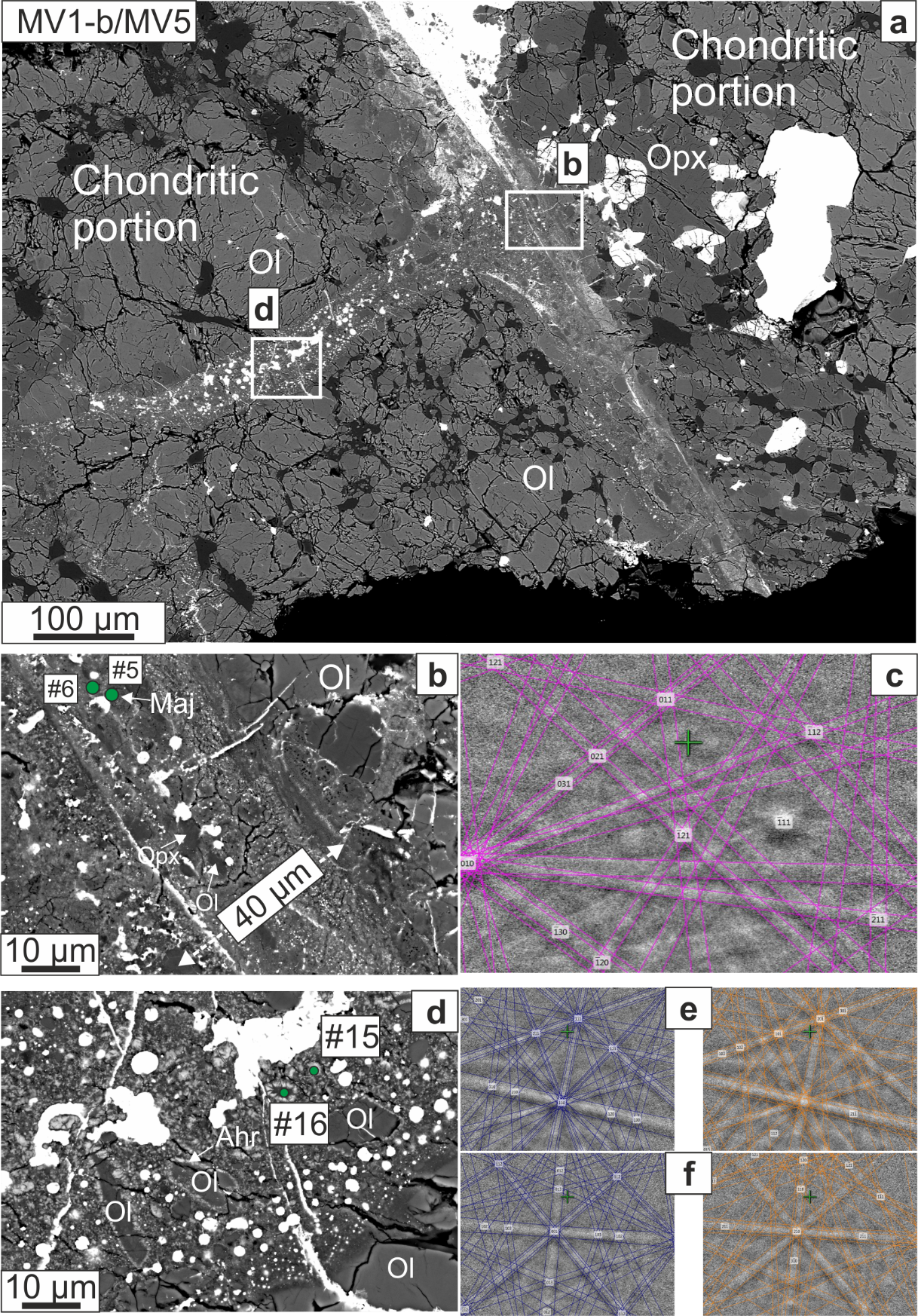


**Supplementary Figure 2:** *Same as in figure 3 annotated for EPMA spots (green coloured circles). Mineral abbreviations: Maj: majorite; Ahr: ahrensite.*


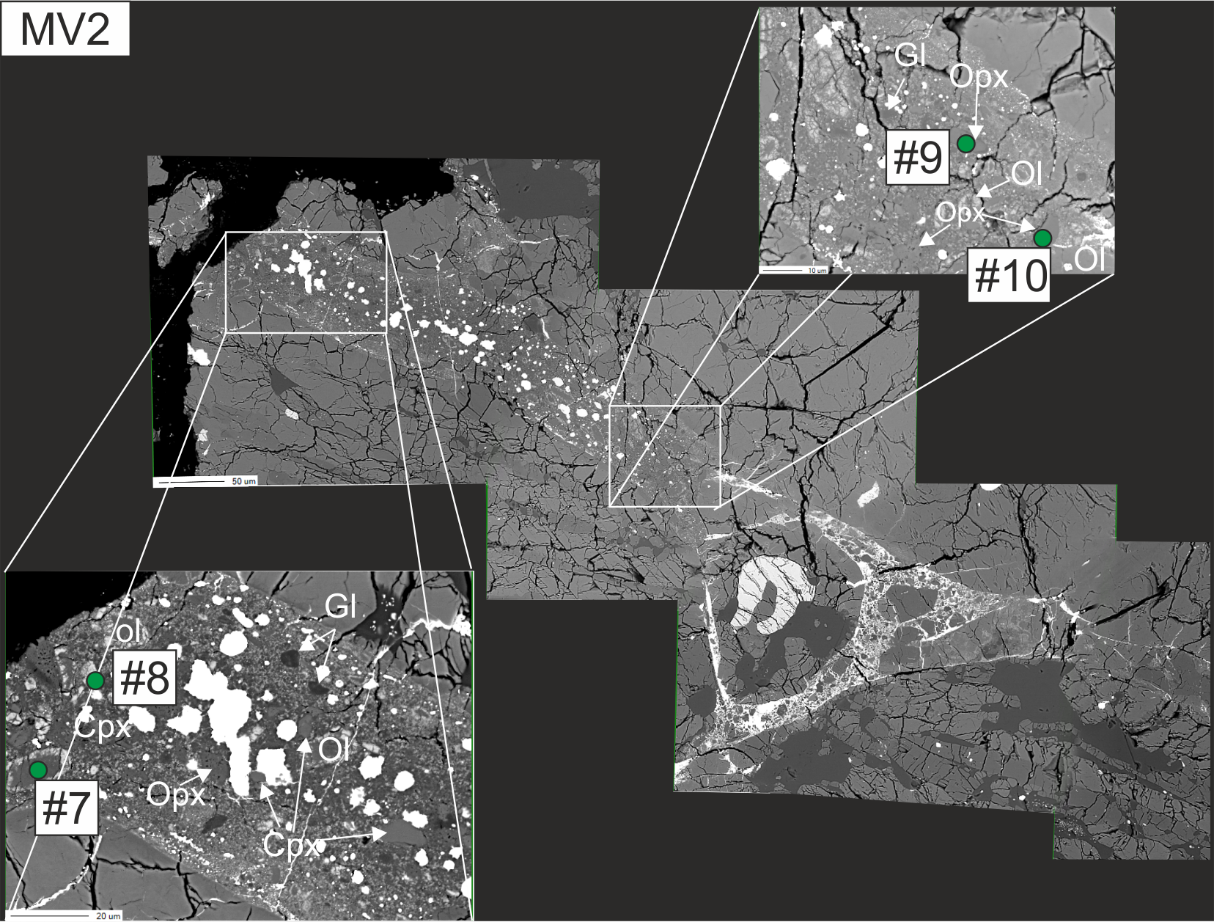


**Supplementary Figure 3: *BSE images*** *of MV2, a melt vein ~50 μm wide apparently devoid of high-pressure minerals annotated for EPMA spots, too (green coloured circles). Mineral abbreviations: Gl: glass.*


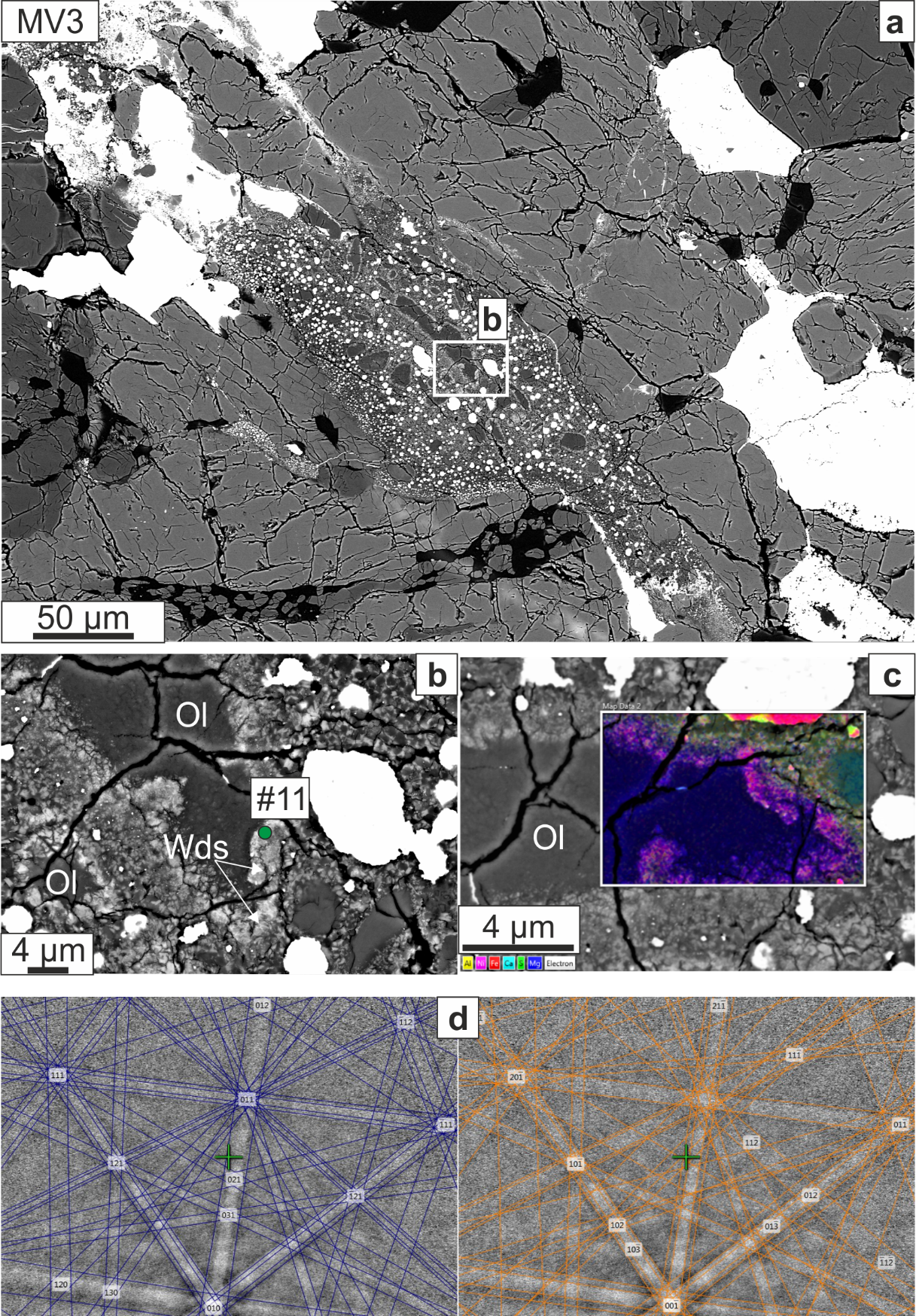


**Supplementary Figure 4:** *Same as in figure 4 annotated for EPMA spots (green coloured circles). Mineral abbreviations: Wds: wadsleyite.*


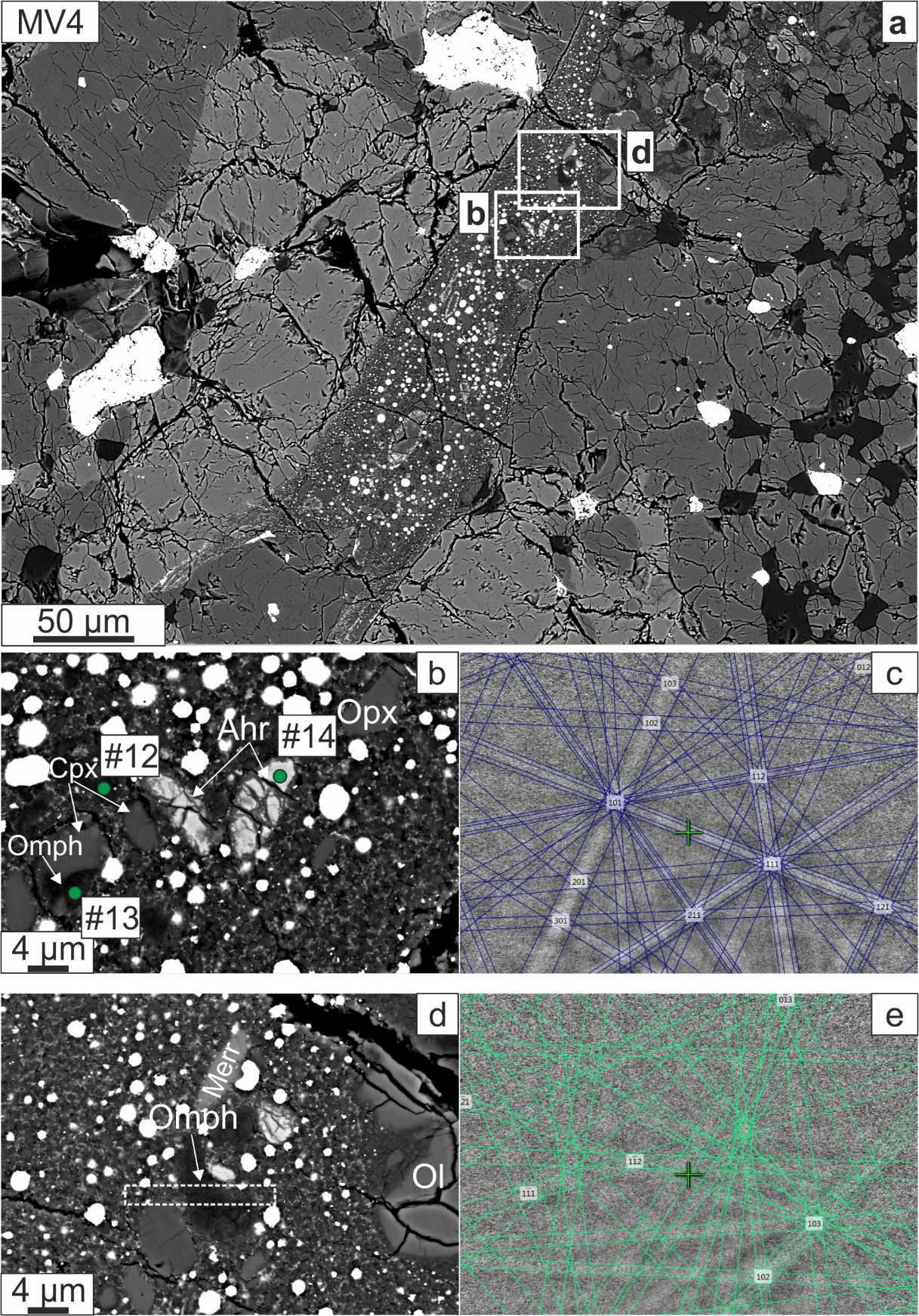


**Supplementary Figure 5:** *Same as in figure 5 annotated for EPMA spots (green coloured circles).* Mineral abbreviations: Merr: merrilite.
